# Changes in triglyceride-glucose index and progression of coronary artery calcification in asymptomatic adults: a longitudinal cohort study

**DOI:** 10.3389/fendo.2026.1976562

**Published:** 2026-10-08

**Authors:** Min Jeong Kim, Yoon Ji Kim, Jae-Heon Kang, Ye Seul Bae

**Affiliations:** 1 Big Data Research Center, Division of Healthcare Planning, Kangbuk Samsung Hospital, Sungkyunkwan University School of Medicine, Seoul, Republic of Korea; 2 Department of Family Medicine, Kangbuk Samsung Hospital, Sungkyunkwan University School of Medicine, Seoul, Republic of Korea; 3 Division of Healthcare Planning, Kangbuk Samsung Hospital, Sungkyunkwan University School of Medicine, Seoul, Republic of Korea; 4 Institute for Clinical Nutrition, Sungkyunkwan University School of Medicine, Seoul, Republic of Korea; 5 Institute for Future Healthcare, Sungkyunkwan University School of Medicine, Seoul, Republic of Korea

**Keywords:** atherosclerosis, coronary artery calcification, coronary artery calcium score, insulin resistance, triglyceride-glucose index

## Abstract

**Introduction:**

The triglyceride-glucose (TyG) index is a readily available surrogate marker of insulin resistance; however, the association between changes in TyG index and coronary artery calcification (CAC) progression remains unclear.

**Methods:**

This retrospective cohort study included 14,407 Korean adults without cardiovascular disease who underwent two baseline examinations during 2012–2018 and at least three examinations overall. At each baseline examination, TyG index status was classified as low or high using a predefined cutoff, yielding low-to-low (L-L), low-to-high (L-H), high-to-low (H-L), and high-to-high (H-H) groups. CAC progression was defined as a difference of ≥2.5 between the square root-transformed coronary artery calcium score (CACS) of the follow-up and index examination. Cox proportional hazards and linear mixed-effects models were used to estimate hazard ratios (HRs) and 5-year fold changes, respectively.

**Results:**

During a median follow-up of 5.34 years, 4,139 participants experienced CAC progression. Compared with the L-L group, the risk of CAC progression increased across the L-H, H-L, and H-H groups, with adjusted HRs (95% confidence intervals) of 1.07 (0.96–1.19), 1.14 (1.02–1.27), and 1.30 (1.20–1.41), respectively. Associations differed between CAC subgroups (*p* for interaction <0.001), with more pronounced associations observed among participants with CACS of zero at both the first and index examinations. The 5-year fold change was 11% higher in the L-H group than in the L-L group and 9% lower in the H-L group than in the H-H group.

**Conclusion:**

Changes in TyG index status were associated with CAC progression, particularly among individuals without detectable CAC. Serial TyG index assessment may aid in cardiovascular risk stratification.

## Graphical Abstract

**Figure:**
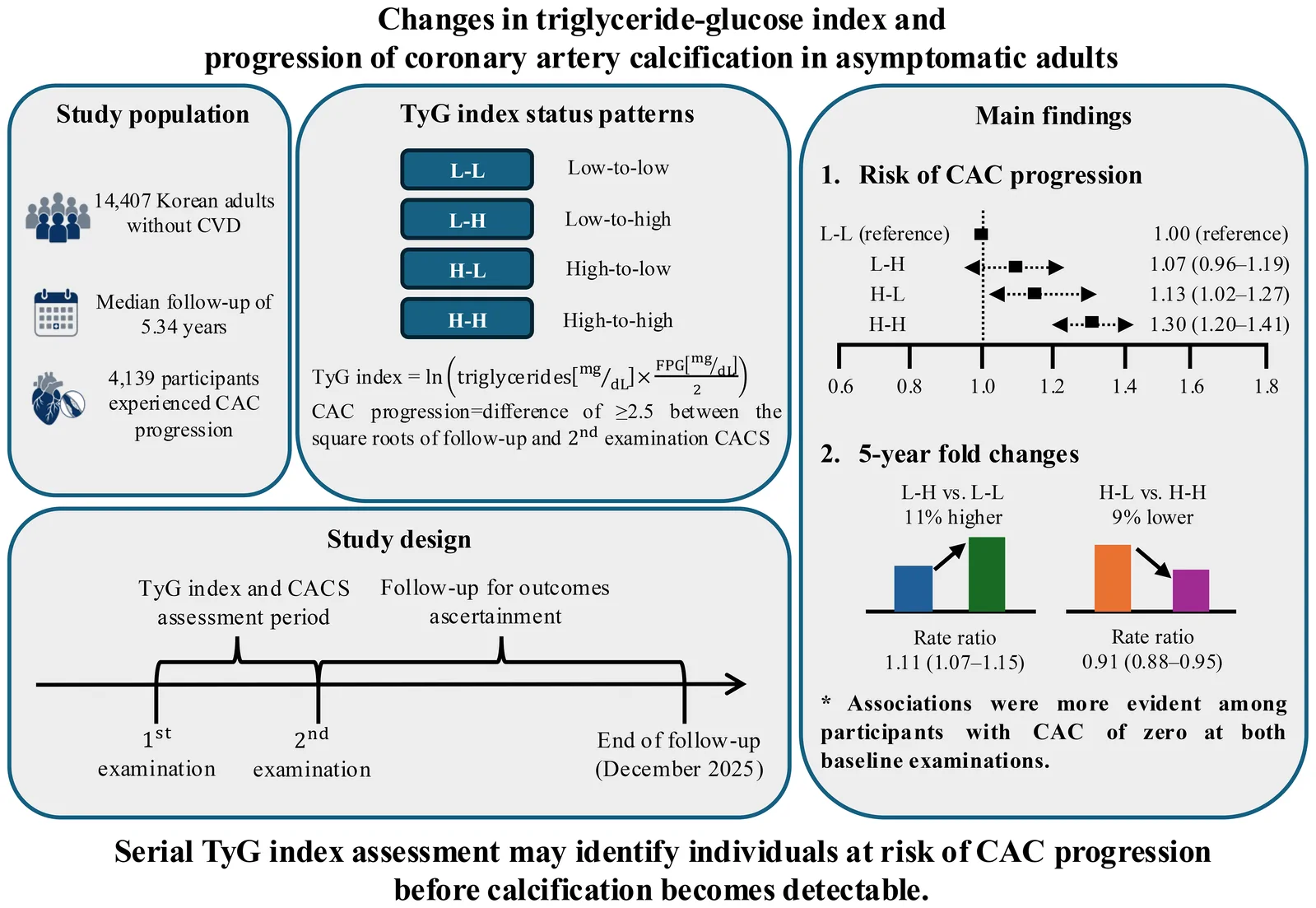

## 1 Introduction

Cardiovascular disease (CVD) remains the leading cause of morbidity and mortality worldwide and continues to impose a substantial public health burden (1). Atherosclerosis, the underlying pathological process of atherosclerotic cardiovascular disease, often develops over a prolonged subclinical period before manifesting as overt cardiovascular events (2, 3). Coronary artery calcification (CAC) is an established marker of subclinical coronary atherosclerosis. The coronary artery calcium score (CACS), measured using computed tomography (CT), provides incremental information for cardiovascular risk assessment beyond traditional cardiovascular risk factors (4, 5). Moreover, beyond a single baseline CACS measurement, CAC progression is associated with an increased risk of subsequent cardiovascular events (6).

Insulin resistance plays an important role in the development of CVD, and several previous studies have shown a relationship between insulin resistance and CAC (7–11). The hyperinsulinemic euglycemic clamp is the gold standard for assessing insulin resistance but is impractical for routine clinical practice and large-scale studies because of its complexity and cost (12). Although the homeostasis model assessment of insulin resistance (HOMA-IR) is widely used as an alternative, it requires fasting insulin measurements, which are inappropriate in primary care settings (13). The triglyceride-glucose (TyG) index, calculated using fasting triglyceride and fasting plasma glucose (FPG) levels, has been proposed as a simple and readily available surrogate marker of insulin resistance (14–16). This index has been shown to correlate closely with both hyperinsulinemic euglycemic clamp-derived insulin sensitivity and HOMA-IR (17–19). Furthermore, the TyG index has demonstrated superior performance to HOMA-IR in the assessment of insulin resistance (20) and the prediction of incident diabetes (21). The TyG index has also shown prognostic value in patients with established cardiovascular disease (22). Previous studies have demonstrated that a higher TyG index is associated with the presence and progression of CAC (15, 23–25), and these associations are particularly evident among individuals with no or mild-to-moderate CAC at baseline (24, 25).

However, evidence remains limited regarding the association between the TyG index and CAC progression while accounting for differences in follow-up duration, particularly across different levels of baseline CAC burden, as previous studies have primarily examined whether CAC progression occurred during follow-up rather than time to progression. Moreover, an inherent limitation of these previous studies is that the TyG index was assessed only at baseline, without considering individual changes over time or their longitudinal implications. Accordingly, serial assessment of the TyG index may improve prognostic stratification by distinguishing persistent elevation from an increase or decrease over time.

Therefore, we aimed to quantify the association between changes in TyG index status and subsequent CAC progression in a large cohort of adults without CVD. We further examined whether this association differed according to CAC status during the exposure-assessment period and evaluated longitudinal changes in CACS in relation to changes in TyG index status.

## 2 Materials and methods

### 2.1 Study population and design

The Kangbuk Samsung Health Study (KSHS) is a large-scale retrospective cohort study of Korean adults who received comprehensive health examinations annually or biennially at Kangbuk Samsung Hospital Health Screening Centers in Seoul or Suwon, Republic of Korea (26). A total of 20,897 Korean adults who underwent at least two baseline health examinations between 2012 and 2018 and at least three examinations in total by December 2025 were initially identified and screened for eligibility. The first and second examinations constituted the exposure-assessment period during which changes in TyG index status were determined. Because classification of TyG index status transition groups required measurements from both examinations, the second examination was designated as the index examination and the baseline for subsequent CAC outcome follow-up. Participants were excluded if they had a history or evidence of CVD at either of the first two examinations, had missing key covariates, or had less than 2 years of follow-up after the index examination. The final analytic sample comprised 14,407 participants. The participant-selection process and study timeline are presented in **Figure 1**.

**Figure 1:**
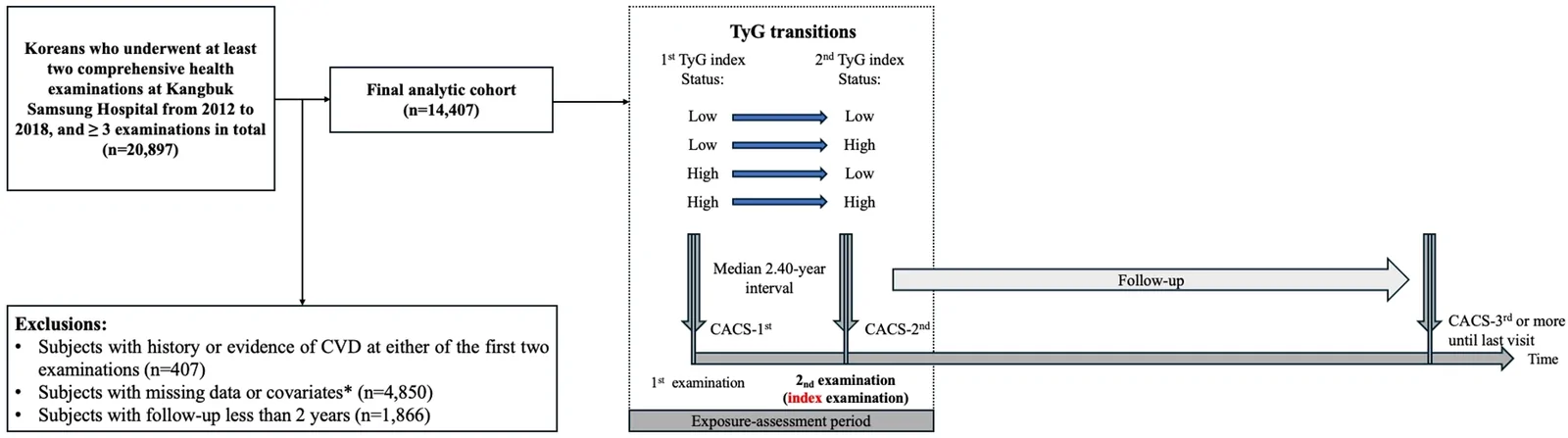
Study population and study design timeline. The triglyceride-glucose (TyG) index was assessed at the first and index baseline examinations conducted between 2012 and 2018. At each examination, TyG index status was classified as low or high using a predefined cutoff, and participants were categorized into four groups according to changes in TyG index status between the two examinations: low-to-low (L-L), low-to-high (L-H), high-to-low (H-L), and high-to-high (H-H). The median interval between the first and index examinations was 2.40 years. Follow-up began at the index examination and continued until the last available examination, with a median follow-up duration of 5.34 years. *Covariates: age, sex, alcohol consumption, physical activity, smoking status, education, body mass index, waist circumference, systolic blood pressure, diastolic blood pressure, fasting blood glucose, glycated hemoglobin, triglycerides, total cholesterol, high-density lipoprotein cholesterol, low-density lipoprotein cholesterol, homeostatic model assessment for insulin resistance, uric acid, estimated glomerular filtration rate, aspartate aminotransferase, alanine aminotransferase, hypertension, dyslipidemia, diabetes.

This study was approved by the Institutional Review Board of Kangbuk Samsung Hospital, which waived the requirement for informed consent because we used only de-identified data routinely collected as part of health screening examinations (IRB No. 2026-04-022). The study was conducted in accordance with the Declaration of Helsinki and relevant institutional regulations.

### 2.2 Data collection

All examinations were performed at Kangbuk Samsung Hospital Health Screening Centers in Seoul and Suwon. At each visit, information on demographic characteristics, including age, sex, and educational level, as well as health-related behaviors, including smoking, alcohol consumption, physical activity, medical history, and medication use, was collected using a self-administered questionnaire as part of the health examination (27).

Educational level was dichotomized as university-level education or higher versus less than university-level education. For smoking status, individuals were classified as never, former, or current smokers. Alcohol consumption was categorized using sex-specific thresholds of <20 vs. ≥ 20 g/day in men and <10 vs. ≥ 10 g/day in women (28). Physical activity was assessed using the validated Korean version of the International Physical Activity Questionnaire (IPAQ) Short Form and grouped into low, moderate, or high levels (29, 30).

Height, weight, and waist circumference (WC) were measured by trained nurses while the participants wore light clothing. WC was measured midway between the lower rib and iliac crest. Body mass index (BMI) was calculated as weight (kg) divided by height squared (m^2^). Blood pressure was measured with participants in a seated position after at least 5 minutes of rest. All blood samples were obtained from the antecubital vein after a fasting period of at least 10 hours. Fasting blood measurements included FPG (mg/dL), glycated hemoglobin (HbA1c) (%), total cholesterol (mg/dL), high-density lipoprotein cholesterol (HDL-C) (mg/dL), low-density lipoprotein cholesterol (LDL-C) (mg/dL), triglycerides (mg/dL), aspartate aminotransferase (AST) (IU/L), alanine aminotransferase (ALT) (IU/L), serum uric acid (mg/dL), insulin (μU/mL), and serum high-sensitivity C-reactive protein (hsCRP) (mg/dL). Insulin resistance was estimated using HOMA-IR, calculated as insulin (μU/mL)×FPG (mg/dL)/405. Serum creatinine levels were measured using the kinetic alkaline picrate (Jaffe) method on a Modular D2400 (Roche Diagnostics, Tokyo, Japan), and kidney function was assessed by calculating the estimated glomerular filtration rate (eGFR) using the four-variable Modification of Diet in Renal Disease Study equation. Serum hsCRP levels were measured using a particle-enhanced immunoturbidimetric assay on a Modular Analytics P800 analyzer (Roche Diagnostics, Tokyo, Japan). Body composition, including fat mass (kg) and skeletal muscle mass (kg), was assessed after an overnight fast using an InBody 720 bioelectrical impedance analyzer (Biospace Inc., Seoul, Korea). The fat mass index and skeletal muscle mass index were calculated by dividing fat mass and skeletal muscle mass in kilograms, respectively, by height squared in meters (31). Electrocardiographic (ECG)-gated CT scans were performed at Seoul and Suwon screening centers using a Lightspeed VCT XTe-64 slice multidetector computed tomography scanner (GE Healthcare, Tokyo, Japan). A standardized ECG-gated acquisition protocol was applied at both screening centers, with a slice thickness of 2.5 mm, rotation time of 400 ms, tube voltage of 120 kV, and tube current-time product of 124 mAs (310 mA × 0.4 s). CACS was calculated using the Agatston method (32). All study data described above were extracted and processed using the Samsung Medical Center Unified Big-data Engine (S-CUBE), an integrated clinical data warehouse and analytics platform of Kangbuk Samsung Hospital.

### 2.3 Definitions

The TyG index was computed as ln[fasting triglycerides (mg/dL) × FPG (mg/dL)/2], where ln denotes the natural logarithm (33). Triglyceride and FPG values were used directly without unit conversion. Because no universally established clinical cutoff for the TyG index is currently available, we used an externally derived cutoff of 8.718 previously reported for predicting prevalent metabolic syndrome in Korean adults (34). This cutoff was nearly identical to the median TyG index of 8.725 at the first examination in the present cohort. Accordingly, the TyG index at each of the first two examinations was classified as low (<8.718) or high (≥8.718). Participants were then categorized according to changes in TyG index status between the first two examinations: low-to-low (L-L), low-to-high (L-H), high-to-low (H-L), and high-to-high (H-H).

CAC progression was defined as a difference of ≥2.5 between the square root-transformed CACS from the index examination to follow-up (Δ√ CACS). This threshold was used to identify changes exceeding expected interscan variability (35) and was not intended to represent a clinical event threshold.

Hypertension was defined as systolic blood pressure (SBP) ≥140 mmHg, diastolic blood pressure (DBP) ≥90 mmHg, self-reported history of hypertension, or current use of antihypertensive medication (36). Dyslipidemia was defined as the current use of lipid-lowering medication. Diabetes was defined as FPG level ≥126 mg/dL, HbA1c level ≥ 6.5%, a self-reported history of diabetes, or current use of antidiabetic medication (36, 37).

### 2.4 Statistical analysis

Continuous variables were summarized as mean ± standard deviation or median [interquartile range (IQR)]. Categorical variables were summarized as numbers (percentages). After checking the distribution of the variables, the one-way analysis of variance (ANOVA) or Kruskal-Wallis test was used for continuous variables, and the Chi-square test was used analyze for categorical variables.

Outcome follow-up began on the date of the index examination. Person-time accrued from the index examination until the first follow-up examination at which CAC progression was identified, the last eligible CAC examination, or the end of follow-up, whichever occurred first. Cox proportional hazards regression models were used to estimate hazard ratios (HRs) and 95% confidence intervals (CIs) for CAC progression according to changes in TyG index status. Four models were constructed: (1) a crude model; (2) multivariable-adjusted model 1: adjusted for age and sex; (3) multivariable-adjusted model 2: adjusted for the variables in model 1 plus traditional cardiovascular risk factors (hypertension, dyslipidemia, diabetes, smoking, alcohol consumption), BMI, and hsCRP; and (4) multivariable-adjusted model 3: adjusted for the variables in model 2 plus ln(CACS + 1) at the index examination. Unless otherwise specified, the covariates included in these models were assessed at the index examination.

To determine whether the association between changes in TyG index status and CAC progression varied according to CAC status during the exposure-assessment period, participants were stratified into two CAC subgroups: those with persistently zero CACS, defined as CACS = 0 at both the first and index examinations, and those with any detectable CAC, defined as CACS >0 at either examination. A fully adjusted Cox model including an interaction term between changes in TyG index status and the CAC subgroup was fitted in the overall cohort. The statistical significance of the interaction term was assessed using a likelihood ratio test comparing nested models with and without the interaction term.

We further evaluated the association between changes in TyG index status and longitudinal changes in CACS from the index examination. Linear mixed-effects models with participant-specific random intercepts were fitted using ln(CACS + 1) as the dependent variable because of its right-skewed distribution. Time since the index examination was scaled in 5-year units, and an interaction between time and TyG index status transition group was included. The model was adjusted for age, sex, traditional cardiovascular risk factors, BMI, and hsCRP. Based on the group-specific time coefficients, we then estimated the adjusted 5-year fold changes for each TyG index status transition group. Ratios of these fold changes were calculated for L-H versus L-L and H-L versus H-H. The estimates and their 95% CIs were obtained by exponentiating the corresponding model coefficients.

To account for differences in the initial TyG level, we performed sensitivity analyses additionally adjusting the fully adjusted Cox and linear mixed-effects models for the continuous TyG index measured at the first examination in both the overall cohort and each CAC subgroup. Further sensitivity analyses were performed using the median TyG index at the first examination (8.725) as an alternative cutoff for defining low and high TyG index status.

All statistical analyses were conducted within S-CUBE using R software version 4.5.3 (R Core Team, Vienna, Austria). A two-sided *p* value <0.05 was considered statistically significant.

## 3 Results

### 3.1 Baseline characteristics

The clinical characteristics of the study population are presented in **Table 1**. Participants were stratified into four groups according to changes in TyG index status between the first two examinations. A total of 14,407 participants were included; their mean age was 43.30 years, and 94.8% were men. The median follow-up duration was 5.34 years. The two largest TyG index status transition groups were H-H (39.61%) and L-L (35.19%). The median interval between the first two examinations was 2.40 years. In the study population, detectable CAC was present in 28.04% of participants, and the prevalence of hypertension, dyslipidemia, diabetes, current smoking, and heavy drinking was 17.9%, 7.9%, 7.8%, 23.8%, and 20.5%, respectively. Compared with participants with persistently low TyG index status, those with persistently high TyG index status had the least favorable cardiometabolic profile, with higher blood pressure, BMI, WC, FPG, HbA1c, triglyceride, total cholesterol, LDL-C, HOMA-IR, and uric acid levels, and lower HDL-C levels. They were also more likely to be current smokers or heavy drinkers and to have hypertension, dyslipidemia, or diabetes. Participants in the L-H and H-L groups generally exhibited intermediate characteristics. The clinical characteristics according to CAC progression are described in **Supplementary Table S1**.

**Table 1:** Baseline characteristics.

Variables	Total (n=14,407)	Changes in TyG index status				*p*	ASD
		L-L (n=5,071)	L-H (n=2,069)	H-L (n=1,560)	H-H (n=5,707)		
Age, years	43.30 (5.41)	42.90 (5.61)	42.99 (5.37)	43.97 (5.58)	43.58 (5.15)	<.001	0.117
Men, %	13,654 (94.77)	4,537 (89.47)	2,002 (96.76)	1,515 (97.12)	5,600 (98.13)	<.001	0.190
Smoking Status, %						<.001	0.225
Never smoker	2,632 (18.26)	1,305 (25.73)	378 (18.27)	232 (14.87)	717 (12.56)		
Former smoker	8,339 (57.88)	2,888 (57.00)	1,230 (59.45)	953 (61.09)	3,268 (57.26)		
Current smoker	3,436 (23.85)	878 (17.31)	461 (22.28)	375 (24.04)	1,722 (30.17)		
Alcohol Consumption, %						<.001	0.132
Heavy drinker	2,949 (20.47)	767 (15.13)	412 (19.91	338 (21.67)	1,432 (25.09)		
Physical Activity						<.001	0.160
Low	6,475 (44.94)	2,148 (42.36)	977 (47.22)	615 (39.42)	2,735 (47.92)		
Moderate	5,672 (39.37)	1,977 (39.00)	801 (38.71)	607 (38.91)	2,287 (40.07)		
High	2,260 (15.69)	946 (18.66)	291 (14.06)	338 (21.67)	685 (12.00)		
Education, %						<.001	0.053
University or more	12,763 (88.59)	4,584 (90.40)	1,828 (88.35)	1,365 (87.50)	4,986 (87.37)		
SBP, mmHg	114.75 (11.81)	111.77 (11.21)	115.71 (11.45)	114.22 (11.69)	117.18 (11.90)	<.001	0.256
DBP, mmHg	75.79 (9.52)	73.15 (9.10)	76.65 (9.16)	75.07 (9.39)	78.02 (9.44)	<.001	0.291
BMI, kg/m^2^	25.20 (3.10)	23.95 (2.86)	25.41 (2.97)	25.08 (2.84)	26.27 (3.01)	<.001	0.417
WC, cm	87.25 (8.16)	83.66 (7.91)	88.02 (7.66)	86.88 (7.38)	90.27 (7.47)	<.001	0.458
FMI	10.97 (3.49)	9.74 (3.22)	11.26 (3.41)	10.64 (3.30)	12.04 (3.44)	<.001	0.375
SMI	30.69 (3.24)	29.70 (3.46)	30.85 (3.04)	30.77 (2.96)	31.50 (2.97)	<.001	0.291
FPG, mg/dL	100.57 (17.39)	94.70 (8.61)	101.00 (15.49)	97.64 (11.00)	106.42 (22.59)	<.001	0.418
HbA1c, %	5.65 (0.60)	5.48 (0.33)	5.62 (0.57)	5.62 (0.50)	5.82 (0.76)	<.001	0.304
Triglycerides, mg/dL	130.00 [92.00, 186.00]	86.00 [68.00, 103.00]	156.00 [137.00, 184.00]	101.00 [85.75, 114.00]	195.00 [157.00, 256.00]	<.001	1.337
Total cholesterol, mg/dL	201.83 (35.43)	192.72 (31.56)	205.93 (32.97)	196.30 (36.25)	209.95 (37.09)	<.001	0.297
HDL-C, mg/dL	52.98 (13.60)	60.85 (14.18)	50.89 (10.93)	54.86 (11.76)	46.24 (10.22)	<.001	0.667
LDL-C, mg/dL	136.15 (32.71)	128.33 (29.98)	141.58 (30.93)	135.32 (34.08)	141.36 (33.85)	<.001	0.240
HOMA-IR	1.71 [1.15, 2.54]	1.23 [0.87, 1.75]	1.87 [1.39, 2.56]	1.44 [1.01, 2.09]	2.27 [1.62, 3.29]	<.001	0.555
Uric acid, mg/dL	6.18 (1.31)	5.80 (1.23)	6.21 (1.25)	6.20 (1.28)	6.50 (1.32)	<.001	0.278
eGFR, ml/min/1.73m^2^	96.32 (15.62)	96.32 (14.98)	96.44 (15.34)	94.24 (15.02)	96.84 (16.37)	<.001	0.086
AST	24.52 (13.86)	22.44 (13.97)	24.55 (13.16)	23.72 (13.69)	26.57 (13.77)	<.001	0.161
ALT	30.57 (22.77)	24.11 (17.62)	31.87 (21.95)	28.19 (25.58)	36.50 (24.60)	<.001	0.306
hsCRP, mg/dL	0.06 [0.03, 0.10]	0.04 [0.03, 0.08]	0.06 [0.04, 0.10]	0.05 [0.03, 0.10]	0.07 [0.04, 0.12]	<.001	0.051
Traditional risk factors							
Hypertension	2,572 (17.85)	591 (11.65)	331 (16.00)	301 (19.29)	1,349 (23.64)	<.001	0.174
Dyslipidemia	1,136 (7.89)	244 (4.81)	123 (5.94)	167 (10.71)	602 (10.55)	<.001	0.139
Diabetes	1,125 (7.81)	108 (2.13)	107 (5.17)	132 (8.46)	778 (13.63)	<.001	0.246
TyG index							
First examination	8.75 (0.57)	8.24 (0.31)	8.46 (0.22)	9.00 (0.25)	9.25 (0.41)	<.001	1.958
Second examination	8.80 (0.58)	8.26 (0.31)	9.00 (0.24)	8.45 (0.23)	9.29 (0.42)	<.001	1.956
CACS	0.00 [0.00, 1.00]	0.00 [0.00, 0.00]	0.00 [0.00, 0.00]	0.00 [0.00, 7.00]	0.00 [0.00, 5.00]	<.001	0.098

Continuous variables are summarized as median [IQR] or mean (SD), and categorical variables are summarized as numbers (%). *p* values were calculated using one-way ANOVA or Kruskal-Wallis test for continuous variables and chi-squared test for categorical variables.

ASD: Absolute standardized difference, IQR: Interquartile range, SD: Standard deviation, ANOVA: Analysis of variance, SBP: Systolic blood pressure, DBP: Diastolic blood pressure, BMI: Body mass index, WC: Waist circumference, FMI: Fat mass index, SMI: Skeletal muscle mass index, FPG: Fasting plasma glucose, HbA1c: Glycated hemoglobin, HDL-C: High-density lipoprotein cholesterol, LDL-C: Low-density lipoprotein cholesterol, HOMA-IR: Homeostasis model assessment of insulin resistance, eGFR: Estimated glomerular filtration rate, AST: Aspartate aminotransferase, ALT: Alanine aminotransferase, hsCRP: high-sensitivity C-reactive protein, TyG: Triglyceride-glucose, CACS: Coronary artery calcium score.

### 3.2 Risk of CAC progression according to changes in TyG index status

#### 3.2.1 Overall cohort

**Table 2** shows the associations between changes in TyG index status and the risk of CAC progression in the overall cohort. During 79,576.07 person-years (PY) of follow-up, 4,139 participants experienced CAC progression. The incidence rate of CAC progression increased across the four groups, from 374.21 per 10,000 PY in the L-L group to 464.85, 585.09, and 657.51 per 10,000 PY in the L-H, H-L, and H-H groups, respectively.

**Table 2:** Risk of CAC progression according to changes in TyG index status in the overall cohort.

Changes in TyG index status	N	Events (N)	Duration (PY)	Incidence rate (per 10^4^ PY)	Crude HR (95% CI)	Adjusted HR (95% CI)		
						Model 1^a^	Model 2^b^	Model 3^c^
L-L	5,071	1,074	28,700.43	374.21	Reference	Reference	Reference	Reference
L-H	2,069	526	11,315.52	464.85	1.31 (1.18, 1.45)	1.25 (1.12, 1.39)	1.13 (1.02, 1.25)	1.07 (0.96, 1.19)
H-L	1,560	502	8,579.82	585.09	1.61 (1.45, 1.79)	1.44 (1.29, 1.60)	1.28 (1.15, 1.42)	1.14 (1.02, 1.27)
H-H	5,707	2,037	30,980.30	657.51	1.84 (1.70, 1.98)	1.66 (1.54, 1.79)	1.34 (1.24, 1.46)	1.30 (1.20, 1.41)

a Model 1 was adjusted for age and sex. ^b^Model 2 was adjusted for the variables in Model 1, traditional risk factors (hypertension, dyslipidemia, diabetes, smoking, alcohol consumption), BMI, and hsCRP. ^c^Model 3 was adjusted for the variables in Model 2 and natural log (CACS + 1) at the index examination.

CAC: Coronary artery calcification, PY: Person-years, HR: Hazard ratio, CI: Confidence interval, BMI: Body mass index, hsCRP: high-sensitivity C-reactive protein, TyG: Triglyceride-glucose, CACS: Coronary artery calcium score.

In the fully adjusted model, compared with the L-L group, the HRs (95% CIs) for the L-H, H-L, and H-H groups were 1.07 (0.96–1.19), 1.14 (1.02–1.27), and 1.30 (1.20–1.41), respectively. The association for the L-H group was attenuated and no longer statistically significant after additional adjustment for ln(CACS + 1) at the index examination, whereas the H-L and H-H groups remained significantly associated with an increased risk of CAC progression.

#### 3.2.2 CAC subgroups

The association between changes in TyG index status and CAC progression differed significantly between CAC subgroups (*p* for interaction <0.001) (**Table 3**). Consistent with the findings in the overall cohort, the incidence rate of CAC progression increased across the four groups within both CAC subgroups. Among participants with persistently zero CACS, compared with the L-L group, the L-H, H-L, and H-H groups were significantly associated with an increased risk of CAC progression after multivariable adjustment, with HRs (95% CIs) of 1.28 (1.06–1.54), 1.38 (1.14–1.68), and 1.53 (1.33–1.77), respectively. In contrast, among participants with detectable CAC, neither L-H nor H-L was significantly associated with CAC progression (HR, 1.04; 95% CI, 0.91–1.18 and HR, 1.05; 95% CI, 0.92–1.19, respectively). However, the H-H group showed a significantly increased risk of CAC progression (HR, 1.12; 95% CI, 1.02–1.24). These associations remained consistent after adjusting for age, sex, traditional cardiovascular risk factors, BMI, and hsCRP.

**Table 3:** Risk of CAC progression according to changes in TyG index status stratified by CAC subgroup.

CAC subgroup	Changes in TyG index status	N	Events (N)	Duration (PY)	Incidence rate (per 10^4^ PY)	Crude HR (95% CI)	Adjusted HR (95% CI)	
							Model 1^a^	Model 2^b^
Persistently zero CACS	Total	10,366	1,298	60,070.64	216.08	–	–	–
	L-L	3,935	347	23,064.60	150.45	Reference	Reference	Reference
	L-H	1,547	173	8,770.29	197.26	1.44 (1.20, 1.73)	1.39 (1.15, 1.66)	1.28 (1.06, 1.54)
	H-L	1,051	147	6,135.69	239.58	1.63 (1.35, 1.98)	1.52 (1.25, 1.84)	1.38 (1.14, 1.68)
	H-H	3,833	631	22,100.06	285.52	1.96 (1.72, 2.24)	1.81 (1.59, 2.07)	1.53 (1.33, 1.77)
Detectable CAC	Total	4,041	2,841	19,505.43	1,456.51	–	–	–
	L-L	1,136	727	5,635.83	1,289.96	Reference	Reference	Reference
	L-H	522	353	2,545.23	1,386.91	1.08 (0.95, 1.23)	1.08 (0.95, 1.23)	1.04 (0.91, 1.18)
	H-L	509	355	2,444.13	1,452.46	1.13 (1.00, 1.29)	1.11 (0.98, 1.27)	1.05 (0.92, 1.19)
	H-H	1,874	1,406	8,880.24	1,583.29	1.28 (1.17, 1.40)	1.25 (1.14, 1.37)	1.12 (1.02, 1.24)

a Model 1 was adjusted for age and sex. ^b^Model 2 was adjusted for the variables in Model 1, traditional risk factors (hypertension, dyslipidemia, diabetes, smoking, alcohol consumption), BMI, and hsCRP.

Subgroup-specific HRs and 95% CIs were estimated separately within the persistently zero CACS and detectable CAC groups. The global *P* value for interaction was obtained using a likelihood ratio test comparing Model 2 with and without an interaction term between TyG index status transition group and CAC status during the exposure-assessment period (*P* for interaction <0.001).

CAC: Coronary artery calcification, PY: Person-years, HR: Hazard ratio, CI: Confidence interval, BMI: Body mass index, hsCRP: high-sensitivity C-reactive protein, TyG: Triglyceride-glucose, CACS: Coronary artery calcium score.

### 3.3 Longitudinal changes in CACS according to changes in TyG index status

#### 3.3.1 Overall cohort

**Table 4** shows the longitudinal changes in CACS according to changes in TyG index status. The estimated 5-year fold changes (95% CIs) were 1.05 (1.02–1.09), 1.16 (1.12–1.21), 1.26 (1.20–1.31), and 1.38 (1.34–1.42) in the L-L, L-H, H-L, and H-H groups, respectively. Compared with the L-L group, the L-H group had an 11% higher 5-year fold change (ratio, 1.11; 95% CI, 1.07–1.15). Conversely, compared with the H-H group, the H-L group had a 9% lower 5-year fold change (ratio, 0.91; 95% CI, 0.88–0.95).

**Table 4:** Model-estimated 5-year fold changes in CACS according to changes in TyG index status in the overall cohort.

Changes in TyG index status	N	5-year fold change (95% CI)	Ratio of 5-year fold changes (95% CI)
L-L	5,071	1.05 (1.02, 1.09)	Reference
L-H	2,069	1.16 (1.12, 1.21)	1.11 (1.07, 1.15)
H-H	5,707	1.38 (1.34, 1.42)	Reference
H-L	1,560	1.26 (1.20, 1.31)	0.91 (0.88, 0.95)

5-year fold changes and ratios of 5-year fold changes were estimated using linear mixed models with random intercepts, with natural log (CACS + 1) as the outcome. The model was adjusted for age, sex, traditional risk factors (hypertension, dyslipidemia, diabetes, smoking, alcohol consumption), BMI, and hsCRP. The L-H group was compared with the L-L group, and the H-L group was compared with the H-H group; L-L and H-H served as the respective reference groups.

CAC: Coronary artery calcification, CI: Confidence interval, BMI: Body mass index, hsCRP: high-sensitivity C-reactive protein, TyG: Triglyceride-glucose.

#### 3.3.2 CAC subgroups

**Table 5** shows the longitudinal changes in CACS according to changes in TyG index status within each CAC subgroup. Among participants with persistently zero CACS, the estimated 5-year fold changes (95% CIs) were 1.35 (1.31–1.38), 1.47 (1.42–1.53), 1.57 (1.50–1.64), and 1.74 (1.69–1.78) in the L-L, L-H, H-L, and H-H groups, respectively. Compared with the L-L group, the L-H group had a 9% higher 5-year fold change (ratio, 1.09; 95% CI, 1.05–1.14). Compared with the H-H group, the H-L group had a 10% lower 5-year fold change (ratio, 0.90; 95% CI, 0.85–0.95).

**Table 5:** Model-estimated 5-year fold changes in CACS according to changes in TyG index status stratified by CAC subgroup.

CAC subgroup	Changes in TyG index status	N	5-year fold change (95% CI)	Ratio of 5-year fold changes (95% CI)
Persistently zero CACS	L-L	3,935	1.35 (1.31, 1.38)	Reference
	L-H	1,547	1.47 (1.42, 1.53)	1.09 (1.05, 1.14)
	H-H	3,833	1.74 (1.69, 1.78)	Reference
	H-L	1,051	1.57 (1.50, 1.64)	0.90 (0.85, 0.95)
Detectable CAC	L-L	1,136	2.13 (2.03, 2.24)	Reference
	L-H	522	2.25 (2.12, 2.39)	1.05 (1.00, 1.12)
	H-H	1,874	2.44 (2.33, 2.55)	Reference
	H-L	509	2.25 (2.12, 2.40)	0.93 (0.88, 0.98)

5-year fold changes and ratios of 5-year fold changes were estimated using linear mixed models with random intercepts, with natural log (CACS + 1) as the outcome. The model was adjusted for age, sex, traditional risk factors (hypertension, dyslipidemia, diabetes, smoking, alcohol consumption), BMI, and hsCRP. The L-H group was compared with the L-L group, and the H-L group was compared with the H-H group; L-L and H-H served as the respective reference groups.

CAC: Coronary artery calcification, CI: Confidence interval, BMI: Body mass index, hsCRP: high-sensitivity C-reactive protein, TyG: Triglyceride-glucose.

Among participants with detectable CAC, the estimated 5-year fold changes (95% CIs) were 2.13 (2.03–2.24), 2.25 (2.12–2.39), 2.25 (2.12–2.40), and 2.44 (2.33–2.55) in the L-L, L-H, H-L, and H-H groups, respectively. The 5-year fold change did not differ significantly between the L-H and L-L groups (ratio, 1.05; 95% CI, 1.00– 1.12). However, compared with the H-H group, the H-L group had a 7% lower 5-year fold change (ratio, 0.93; 95% CI, 0.88–0.98).

### 3.4 Sensitivity analyses

After additional adjustment for the continuous TyG index measured at the first examination, the associations with CAC progression were substantially attenuated in the Cox analyses. In the overall cohort, the adjusted HRs (95% CIs) for the L-H, H-L, and H-H groups versus the L-L group were 1.04 (0.93–1.15), 0.97 (0.85–1.09), and 1.04 (0.93–1.17), respectively (**Supplementary Table S2**). None of the associations remained statistically significant within either CAC subgroup (**Supplementary Table S3**). In contrast, the results of the linear mixed-effects analyses were materially unchanged after additional adjustment for the first-examination TyG index (**Supplementary Tables S4 and S5**).

Analyses using the median-based cutoff also yielded results generally consistent with the primary findings (**Supplementary Tables S6–S9**).

## 4 Discussion

In this study, we investigated the association between changes in TyG index status and subsequent CAC progression in 14,407 Korean adults without CVD. Participants with persistently high TyG index status had the highest risk of CAC progression, whereas those whose TyG index status changed from high to low remained at a higher risk than those with persistently low TyG index status. Notably, the association differed substantially between CAC subgroups. Among participants with persistently zero CACS, the L-H and H-L groups, as well as those with persistently high TyG index status, were associated with an increased risk of CAC progression. In contrast, among participants with detectable CAC, the increased risk was confined to those with persistently high TyG index status. In the analyses of repeated CACS measurements, the estimated 5-year fold change was higher in the L-H group than in the persistently low group and lower in the H-L group than in the persistently high group, with these differences being more pronounced among participants with persistently zero CACS. After additional adjustment for the continuous TyG index measured at the first examination, the associations in the Cox analyses were substantially attenuated, whereas the findings from the linear mixed-effects analyses were materially unchanged. However, because the first-examination TyG index is an inherent component of the TyG index status transition groups, these sensitivity analyses should be interpreted cautiously owing to potential multicollinearity and overadjustment.

Our findings corroborate and extend previous evidence linking an elevated TyG index to subclinical coronary atherosclerosis. Earlier studies have demonstrated that a higher baseline TyG index is associated with prevalent CAC and subsequent CAC progression (15, 23–25). These associations appeared to be more pronounced among individuals without heavy calcification at baseline, suggesting that the TyG index may help identify individuals at higher risk of CAC progression before substantial calcification develops (24, 25). However, these studies assessed the TyG index at a single baseline examination and therefore could not distinguish persistent metabolic abnormalities from changes over time. Evidence from studies using repeated TyG index measurements suggests that longitudinal exposure patterns may provide information beyond a single measurement. A longer duration of higher TyG index has been associated with an increased risk of incident CVD (38), while the temporal pattern of the cumulative TyG index has also been associated with subsequent cardiovascular risk (39). Using TyG index status measured at two examinations, our study extends these findings to CAC progression while capturing changes in TyG index status over time. The consistent results from the time-to-event and repeated-measures analyses further support an association of these longitudinal TyG index patterns with both the occurrence and extent of CAC progression. The distinct associations according to CAC subgroup suggest that the clinical relevance of changes in TyG index status may vary across different stages of subclinical coronary atherosclerosis.

An important finding of our study was the intermediate-risk profile observed in the H-L group. Although participants in this group remained at a higher risk of CAC progression than those with persistently low TyG index status, their estimated 5-year fold change was lower than that of participants with persistently high TyG index status. The residual excess risk observed in the H-L group may be associated with prior exposure to an adverse metabolic state. Previous population-based studies similarly showed that recovery from metabolic syndrome or remission from diabetes was associated with a lower cardiovascular risk than persistent metabolic abnormalities, although the risk remained higher than that among individuals who remained metabolically healthy (40, 41). These observations suggest that cardiovascular risk may not normalize immediately following metabolic improvement. Nevertheless, the lower estimated fold change in the H-L group indicates that a change from high to low TyG index status was associated with a lower subsequent CACS fold change than persistently high TyG index status. This finding is in line with a previous cohort study in which the direction of change in the TyG index over time was associated with incident CVD risk (42).

The substantial heterogeneity between CAC subgroups may reflect differences in the prognostic relevance of the TyG index across stages of subclinical coronary atherosclerosis. A CACS of zero indicates the absence of CT-detectable calcification but does not necessarily exclude earlier noncalcified coronary atherosclerosis (43, 44). Among individuals with persistently zero CACS, differences in CAC progression risk across the four groups suggest that changes in TyG index status may serve as a marker of longitudinal metabolic exposure and may help distinguish individuals with different risks of subsequent calcification before it becomes detectable. Together with previous studies reporting stronger associations between the TyG index and CAC progression in individuals without heavy baseline calcification (24, 25), these findings suggest that individuals with persistently zero CACS should not be regarded as a uniformly low-risk group, but rather as a population in which metabolic risk profiles may further stratify future calcification risk. Once CAC is detectable, however, subsequent CAC progression may be influenced by baseline CAC burden, age, and cumulative cardiovascular risk factors (45, 46), potentially weakening the association between changes in TyG index status and CAC progression events. Nevertheless, findings from repeated-measures analyses suggest that these changes may still provide information on the pace of calcification among individuals with established CAC.

From a clinical perspective, the TyG index is a simple and readily available marker derived from fasting triglyceride and glucose levels, which are routinely measured during health examinations. Consistent with its potential clinical utility, the TyG index has been incorporated into models predicting cardiovascular events in patients with newly diagnosed hypertension and obstructive sleep apnea (47, 48). Accordingly, serial assessment of the TyG index may be useful for identifying individuals who warrant closer monitoring of cardiometabolic risk factors and sustained lifestyle or preventive management, even among those who appear to be at low risk with persistently zero CACS.

This study has several strengths. First, to our knowledge, this is among the first large-scale studies to evaluate longitudinal TyG index status patterns in relation to subsequent CAC progression, extending prior studies that primarily assessed the TyG index at a single baseline examination. This design enabled us to distinguish persistent elevation from directional changes in TyG index status over time. Second, we evaluated CAC progression using both time-to-event and repeated-measures approaches, providing complementary information on whether CAC progression occurred and how rapidly CACS changed over time. Finally, stratified analyses by CAC subgroup provided insight into how the relevance of changes in TyG index status may differ across stages of subclinical coronary atherosclerosis, particularly between individuals without detectable CAC and those with established calcification.

Several limitations should be acknowledged. First, because men comprised the majority of the study population, sex-specific analyses could not be performed. Although sex was adjusted for in the analyses, potential sex differences in the association between changes in TyG index status and CAC progression warrant further investigation. Second, TyG index status was defined based on measurements from the first two examinations, and longer-term trajectories could not be fully captured. Third, although the square root-transformed CACS threshold was designed to distinguish progression from interscan variability, it does not necessarily represent a clinically established threshold for atherosclerotic progression or cardiovascular events. Finally, the study population consisted of relatively healthy Korean adults undergoing regular health screening examinations, which may limit the generalizability of our findings to the general Korean population.

## 5 Conclusion

In conclusion, changes in TyG index status were associated with subsequent CAC progression in Korean adults without CVD. Persistently high TyG index status was associated with the greatest risk of CAC progression, whereas the H-L group showed residual excess risk compared with the persistently low group, but a lower estimated 5-year fold change than that in the persistently high group. The prognostic relevance of changes in TyG index status differed between CAC subgroups, with broader associations observed among individuals without detectable CAC. These findings suggest that serial assessment of the TyG index may provide useful information for evaluating metabolic risk related to subclinical coronary atherosclerosis during repeated health examinations.

## Data Availability

The data analyzed in this study is subject to the following licenses/restrictions: The data that support the findings of this study are not publicly available due to institutional policies and ethical restrictions related to the use of personal health information. However, de-identified data may be made available to qualified researchers upon reasonable request and approval by the Institutional Review Board of Kangbuk Samsung Hospital and the Data Sharing Committee of the Kangbuk Samsung Health Study. Requests to access these datasets should be directed to Ye Seul Bae, byeye1313@gmail.com.
