# Supplementary material for: Changes in triglyceride-glucose index and progression of coronary artery calcification in asymptomatic adults: a longitudinal cohort study
Source: Front Endocrinol (Lausanne). 2026 Oct 8;17:1976562. doi: 10.3389/fendo.2026.1976562 (PMC13686911; doi:10.3389/fendo.2026.1976562)
Supplement: Supplementary file 1 [file Table1.docx]

**Supplementary Table S1. Baseline Characteristics According to CAC Progression**

| Variables | Total  (n=14,407) | No CAC progression  (n=10,268) | CAC progression  (n=4,139) | *p* | ASD |
| --- | --- | --- | --- | --- | --- |
| Age, years | 43.30 (5.41) | 42.61 (5.27) | 45.00 (5.37) | <.001 | 0.451 |
| Men, % | 13,654 (94.77) | 9,594 (93.44) | 4,060 (98.09) | <.001 | 0.233 |
| Smoking Status, % |  |  |  | <.001 | 0.260 |
| Never smoker | 2,632 (18.27) | 2,136 (20.80) | 496 (11.98) |  |  |
| Former smoker | 8,339 (57.88) | 5,884 (57.30) | 2,455 (59.31) |  |  |
| Current smoker | 3,436 (23.85) | 2,248 (21.89) | 1,188 (28.70) |  |  |
| Alcohol Consumption, % |  |  |  | <.001 | 0.129 |
| Heavy drinker | 2,949 (20.47) | 1,946 (18.95) | 1,003 (24.23) |  |  |
| Physical Activity |  |  |  | <.001 | 0.102 |
| Low | 6,475 (44.94) | 4,751 (46.27) | 1,724 (41.65) |  |  |
| Moderate | 5,672 (39.37) | 3,988 (38.84) | 1,684 (40.69) |  |  |
| High | 2,260 (15.69) | 1,529 (14.89) | 731 (17.66) |  |  |
| Education, % |  |  |  | 0.737 | 0.007 |
| University or more | 12,763 (88.59) | 9,090 (88.53) | 3,673 (88.74) |  |  |
| SBP, mmHg | 114.75 (11.81) | 113.80 (11.56) | 117.10 (12.10) | <.001 | 0.279 |
| DBP, mmHg | 75.79 (9.52) | 74.86 (9.34) | 78.09 (9.57) | <.001 | 0.342 |
| BMI, kg/m^2^ | 25.20 (3.10) | 25.01 (3.08) | 25.68 (3.11) | <.001 | 0.218 |
| WC, cm | 87.25 (8.16) | 86.76 (8.20) | 88.48 (7.94) | <.001 | 0.213 |
| FMI | 10.97 (3.49) | 10.85 (3.48) | 11.26 (3.50) | <.001 | 0.117 |
| SMI | 30.69 (3.24) | 30.50 (3.32) | 31.18 (2.98) | <.001 | 0.214 |
| FPG, mg/dL | 100.57 (17.39) | 98.95 (14.56) | 104.57 (22.45) | <.001 | 0.297 |
| HbA1c, % | 5.65 (0.60) | 5.59 (0.52) | 5.79 (0.75) | <.001 | 0.305 |
| Triglycerides, mg/dL | 130.00 [92.00, 186.00] | 125.00 [89.00, 180.00] | 142.00 [100.00, 203.00] | <.001 | 0.179 |
| Total cholesterol, mg/dL | 201.83 (35.43) | 200.78 (34.15) | 204.45 (38.29) | <.001 | 0.101 |
| HDL-C, mg/dL | 52.98 (13.60) | 53.59 (13.77) | 51.49 (13.06) | <.001 | 0.156 |
| LDL-C, mg/dL | 136.15 (32.71) | 135.55 (31.80) | 137.66 (34.81) | <.001 | 0.063 |
| HOMA-IR | 1.71 [1.15, 2.54] | 1.65 [1.11, 2.44] | 1.89 [1.23, 2.76] | <.001 | 0.192 |
| Uric acid, mg/dL | 6.18 (1.31) | 6.13 (1.31) | 6.31 (1.31) | <.001 | 0.135 |
| eGFR, ml/min/1.73m^2^ | 96.32 (15.62) | 96.63 (15.55) | 95.54 (15.76) | <.001 | 0.070 |
| AST | 24.52 (13.86) | 23.90 (12.23) | 26.05 (17.16) | <.001 | 0.144 |
| ALT | 30.57 (22.77) | 29.58 (20.05) | 33.03 (28.25) | <.001 | 0.141 |
| hsCRP, mg/dL | 0.06 [0.03, 0.10] | 0.05 [0.03, 0.10] | 0.06 [0.04, 0.11] | <.001 | 0.024 |
| Traditional risk factors |  |  |  |  |  |
| Hypertension | 2,572 (17.85) | 1,381 (13.45) | 1,191 (28.78) | <.001 | 0.382 |
| Dyslipidemia | 1136 (7.89) | 591 (5.76) | 545 (13.17) | <.001 | 0.255 |
| Diabetes | 1125 (7.81) | 548 (5.34) | 577 (13.94) | <.001 | 0.295 |
| TyG index |  |  |  |  |  |
| First examination | 8.75 (0.57) | 8.69 (0.56) | 8.91 (0.58) | <.001 | 0.387 |
| Second examination | 8.80 (0.58) | 8.75 (0.57) | 8.92 (0.59) | <.001 | 0.283 |
| CACS | 0.00 [0.00, 1.00] | 0.00 [0.00, 0.00] | 9.00 [0.00, 48.50] | <.001 | 0.515 |

Continuous variables are summarized as median [IQR] or mean (SD), and categorical variables are summarized as numbers (%). *p* values were calculated using one-way ANOVA or Kruskal-Wallis test for continuous variables and chi-squared test for categorical variables.
Abbreviations: ASD: Absolute standardized difference, IQR: Interquartile range, SD: Standard deviation, ANOVA: Analysis of variance, SBP: Systolic blood pressure, DBP: Diastolic blood pressure, BMI: Body mass index, WC: Waist circumference, FMI: Fat mass index, SMI: Skeletal muscle mass index, FPG: Fasting plasma glucose, HbA1c: Glycated hemoglobin, HDL-C: High-density lipoprotein cholesterol, LDL-C: Low-density lipoprotein cholesterol, HOMA-IR: Homeostasis model assessment of insulin resistance, eGFR: Estimated glomerular filtration rate, AST: Aspartate aminotransferase, ALT: Alanine aminotransferase, hsCRP: high-sensitivity C-reactive protein, TyG: Triglyceride-glucose, CACS: Coronary artery calcium score.

**Supplementary Table S2. Risk of CAC Progression According to Changes in TyG Index Status After Additional Adjustment for the First-Examination TyG Index in the Overall Cohort**

| Changes in TyG index status | N | Events (N) | Adjusted HR^a^ (95% CI) |
| --- | --- | --- | --- |
|  |  |  |  |
| L-L | 5,071 | 1,074 | Reference |
| L-H | 2,069 | 526 | 1.04 (0.93, 1.15) |
| H-L | 1,560 | 502 | 0.97 (0.85, 1.09) |
| H-H | 5,707 | 2,037 | 1.04 (0.93, 1.17) |

^a^ The model was adjusted for age, sex, hypertension, dyslipidemia, diabetes, smoking, alcohol consumption, BMI, hsCRP, ln(CACS+1) at the index examination, and the continuous TyG index measured at the first examination.
Abbreviations: CAC: Coronary artery calcification, HR: Hazard ratio, CI: Confidence interval, BMI: Body mass index, hsCRP: high-sensitivity C-reactive protein, TyG: Triglyceride-glucose, CACS: Coronary artery calcium score.

**Supplementary Table S3. Risk of CAC Progression According to Changes in TyG Index Status After Additional Adjustment for the First-Examination TyG Index, Stratified by CAC Subgroup**

| CAC subgroup | Changes in TyG index status | N | Events (N) | Adjusted HR (95% CI)^a^ |
| --- | --- | --- | --- | --- |
|  |  |  |  |  |
| Persistently zero CACS | **L-L** | 3,935 | 347 | Reference |
|  | **L-H** | 1,547 | 173 | 1.20 (0.99, 1.44) |
|  | **H-L** | 1,051 | 147 | 1.08 (0.87, 1.35) |
|  | **H-H** | 3,833 | 631 | 1.10 (0.89, 1.35) |
| Detectable CAC | **L-L** | 1,136 | 727 | Reference |
|  | **L-H** | 522 | 353 | 1.01 (0.89, 1.15) |
|  | **H-L** | 509 | 355 | 0.93 (0.80, 1.08) |
|  | **H-H** | 1,874 | 1,406 | 0.96 (0.84, 1.10) |

^a^ The model was adjusted for age, sex, hypertension, dyslipidemia, diabetes, smoking, alcohol consumption, BMI, hsCRP, and the continuous TyG index measured at the first examination.
Subgroup-specific HRs and 95% CIs were estimated separately within the persistently zero CACS and detectable CAC groups. The global *P* value for interaction was obtained using a likelihood ratio test comparing additional adjustment model with and without an interaction term between TyG index status transition group and CAC status during the exposure-assessment period (*P* for interaction <0.001).
Abbreviations: CAC: Coronary artery calcification, HR: Hazard ratio, CI: Confidence interval, BMI: Body mass index, hsCRP: high-sensitivity C-reactive protein, TyG: Triglyceride-glucose, CACS: Coronary artery calcium score.

**Supplementary Table S4. Model-Estimated 5-Year Fold Changes in CACS According to Changes in TyG Index Status in the Overall Cohort After Additional Adjustment for the First-Examination TyG Index**

| Changes in TyG index status | N | 5-year fold change  (95% CI) | Ratio of 5-year fold changes  (95% CI) |
| --- | --- | --- | --- |
| L-L | 5,071 | 1.06  (1.02, 1.09) | Reference |
| L-H | 2,069 | 1.17  (1.12, 1.22) | 1.11  (1.07, 1.15) |
| H-H | 5,707 | 1.39  (1.34, 1.43) | Reference |
| H-L | 1,560 | 1.26  (1.21, 1.32) | 0.91  (0.88, 0.95) |

5-year fold changes and ratios of 5-year fold changes were estimated using linear mixed models with random intercepts, with natural log (CACS+1) as the outcome. The model was adjusted for age, sex, traditional risk factors (hypertension, dyslipidemia, diabetes, smoking, alcohol consumption), BMI, hsCRP, and the continuous TyG index measured at the first examination. The L–H group was compared with the L–L group, and the H–L group was compared with the H–H group; L–L and H–H served as the respective reference groups.
Abbreviations: CAC: Coronary artery calcification, CI: Confidence interval, BMI: Body mass index, hsCRP: high-sensitivity C-reactive protein, TyG: Triglyceride-glucose.

**Supplementary Table S5. Model-Estimated 5-Year Fold Changes in CACS According to Changes in TyG Index Status After Additional Adjustment for the First-Examination TyG Index, Stratified by CAC Subgroup**

| CAC subgroup | Changes in TyG index status | N | 5-year fold change  (95% CI) | Ratio of 5-year fold changes  (95% CI) |
| --- | --- | --- | --- | --- |
| Persistently zero CACS | **L-L** | 3,935 | 1.35  (1.32, 1.38) | Reference |
|  | **L-H** | 1,547 | 1.48  (1.42, 1.54) | 1.09  (1.05, 1.14) |
|  | **H-H** | 3,833 | 1.74  (1.70, 1.79) | Reference |
|  | **H-L** | 1,051 | 1.57  (1.50, 1.64) | 0.90  (0.86, 0.95) |
| Detectable CAC | **L-L** | 1,136 | 2.13  (2.03, 2.24) | Reference |
|  | **L-H** | 522 | 2.25  (2.12, 2.39) | 1.06  (1.00, 1.12) |
|  | **H-H** | 1,874 | 2.44  (2.33, 2.55) | Reference |
|  | **H-L** | 509 | 2.25  (2.12, 2.39) | 0.93  (0.88, 0.98) |

5-year fold changes and ratios of 5-year fold changes were estimated using linear mixed models with random intercepts, with natural log (CACS+1) as the outcome. The model was adjusted for age, sex, traditional risk factors (hypertension, dyslipidemia, diabetes, smoking, alcohol consumption), BMI, hsCRP, and the continuous TyG index measured at the first examination. The L–H group was compared with the L–L group, and the H–L group was compared with the H–H group; L–L and H–H served as the respective reference groups.
Abbreviations: CAC: Coronary artery calcification, CI: Confidence interval, BMI: Body mass index, hsCRP: high-sensitivity C-reactive protein, TyG: Triglyceride-glucose.

**Supplementary Table S6. Risk of CAC Progression According to Changes in TyG Index Status Defined Using Median-Based Cutoff (TyG Index 8.725) in the Overall Cohort**

| Changes in TyG index status | N | Events (N) | Duration (PY) | Incidence rate  (per 10^4^ PY) | Crude HR  (95% CI) | Adjusted HR  (95% CI) | | |
| --- | --- | --- | --- | --- | --- | --- | --- | --- |
|  |  |  |  |  |  | **Model 1^a^** | **Model 2^b^** | **Model 3^c^** |
| L-L | 5,131 | 1,094 | 29,037.33 | 376.76 | Reference | Reference | Reference | Reference |
| L-H | 2,068 | 522 | 11,304.56 | 461.76 | 1.29  (1.16, 1.43) | 1.23  (1.11, 1.37) | 1.12  (1.00, 1.24) | 1.07  (0.96, 1.19) |
| H-L | 1,557 | 501 | 8,557.65 | 585.44 | 1.60  (1.44, 1.78) | 1.43  (1.28, 1.59) | 1.27  (1.14, 1.41) | 1.14  (1.02, 1.27) |
| H-H | 5,651 | 2,022 | 30,676.52 | 659.14 | 1.83  (1.70, 1.97) | 1.66  (1.54, 1.79) | 1.34  (1.24, 1.45) | 1.30  (1.20, 1.40) |

^a^Model 1 was adjusted for age and sex. ^b^Model 2 was adjusted for the variables in Model 1, traditional risk factors (hypertension, dyslipidemia, diabetes, smoking, alcohol consumption), BMI, and hsCRP. ^c^Model 3 was adjusted for the variables in Model 2 and natural log (CACS+1) in the second examination.
Abbreviations: CAC: Coronary artery calcification, PY: Person-years, HR: Hazard ratio, CI: Confidence interval, BMI: Body mass index, hsCRP: high-sensitivity C-reactive protein, TyG: Triglyceride-glucose, CACS: Coronary artery calcium score.

**Supplementary Table S7. Risk of CAC Progression According to Changes in TyG Index Status Defined Using Median-Based Cutoff (TyG Index 8.725) Stratified by CAC Subgroup**

| CAC subgroup | Changes in TyG index status | N | Events (N) | Duration (PY) | Incidence rate  (per 10^4^ PY) | Crude HR  (95% CI) | Adjusted HR  (95% CI) | |
| --- | --- | --- | --- | --- | --- | --- | --- | --- |
|  |  |  |  |  |  |  | **Model 1^a^** | **Model 2^b^** |
| Persistently zero CACS | **L-L** | 3,980 | 356 | 23,324.07 | 152.63 | Reference | Reference | Reference |
|  | **L-H** | 1,546 | 170 | 8,759.07 | 194.08 | 1.40  (1.17, 1.68) | 1.34  (1.12, 1.61) | 1.24  (1.03, 1.49) |
|  | **H-L** | 1,043 | 145 | 6,091.64 | 238.03 | 1.60  (1.32, 1.94) | 1.48  (1.22, 1.80) | 1.35  (1.11, 1.64) |
|  | **H-H** | 3,797 | 627 | 21,895.86 | 286.36 | 1.95  (1.71, 2.22) | 1.80  (1.58, 2.05) | 1.52  (1.32, 1.75) |
| Detectable CAC | **L-L** | 1,151 | 738 | 5,713.26 | 1,291.73 | Reference | Reference | Reference |
|  | **L-H** | 522 | 352 | 2,545.50 | 1,382.84 | 1.08  (0.95, 1.22) | 1.08  (0.95, 1.22) | 1.03  (0.91, 1.17) |
|  | **H-L** | 514 | 356 | 2,466.01 | 1,443.63 | 1.13  (1.00, 1.29) | 1.11  (0.98, 1.26) | 1.05  (0.92, 1.19) |
|  | **H-H** | 1,854 | 1,395 | 8,780.66 | 1,588.72 | 1.28  (1.17, 1.40) | 1.26  (1.15, 1.38) | 1.13  (1.03, 1.24) |

^a^Model 1 was adjusted for age and sex. ^b^Model 2 was adjusted for the variables in Model 1, traditional risk factors (hypertension, dyslipidemia, diabetes, smoking, alcohol consumption), BMI, and hsCRP.
Subgroup-specific HRs and 95% CIs were estimated separately within the persistently zero CACS and detectable CAC groups. The global *P* value for interaction was obtained using a likelihood ratio test comparing Model 2 with and without an interaction term between TyG index status transition group and CAC subgroup (*P* for interaction <0.001).
Abbreviations: CAC: Coronary artery calcification, PY: Person-years, HR: Hazard ratio, CI: Confidence interval, BMI: Body mass index, hsCRP: high-sensitivity C-reactive protein, TyG: Triglyceride-glucose, CACS: Coronary artery calcium score.

**Supplementary Table S8. Model-estimated 5-year changes in CACS According to Changes in TyG Index Status Defined Using Median-Based Cutoff (TyG Index 8.725) in the Overall Cohort**

| Changes in TyG index status | N | 5-year fold change  (95% CI) | Ratio of 5-year fold changes  (95% CI) |
| --- | --- | --- | --- |
| L-L | 5,131 | 1.05  (1.02, 1.09) | Reference |
| L-H | 2,068 | 1.17  (1.12, 1.21) | 1.11  (1.07, 1.15) |
| H-H | 5,651 | 1.38  (1.33, 1.42) | Reference |
| H-L | 1,557 | 1.25  (1.20, 1.31) | 0.91  (0.88, 0.95) |

5-year fold changes and ratios of 5-year fold changes were estimated using linear mixed models with random intercepts, with natural log (CACS+1) as the outcome. The model was adjusted for age, sex, traditional risk factors (hypertension, dyslipidemia, diabetes, smoking, alcohol consumption), BMI, and hsCRP. The L–H group was compared with the L–L group, and the H–L group was compared with the H–H group; L–L and H–H served as the respective reference groups.
Abbreviations: CAC: Coronary artery calcification, CI: Confidence interval, BMI: Body mass index, hsCRP: high-sensitivity C-reactive protein, TyG: Triglyceride-glucose.

**Supplementary Table S9. Model-estimated 5-year CACS According to Changes in TyG Index Status Defined Using Median-Based Cutoff (TyG Index 8.725) Stratified by CAC Subgroup**

| CAC subgroup | Changes in TyG index status | N | 5-year fold change  (95% CI) | Ratio of 5-year fold changes  (95% CI) |
| --- | --- | --- | --- | --- |
| Persistently zero CACS | **L-L** | 3,980 | 1.35  (1.32, 1.39) | Reference |
|  | **L-H** | 1,546 | 1.47  (1.42, 1.53) | 1.09  (1.04, 1.14) |
|  | **H-H** | 3,797 | 1.74  (1.69, 1.78) | Reference |
|  | **H-L** | 1,043 | 1.57  (1.50, 1.64) | 0.90  (0.86, 0.95) |
| Detectable CAC | **L-L** | 1,151 | 2.14  (2.03, 2.24) | Reference |
|  | **L-H** | 522 | 2.26  (2.13, 2.40) | 1.06  (1.0005, 1.12) |
|  | **H-H** | 1,854 | 2.44  (2.33, 2.55) | Reference |
|  | **H-L** | 514 | 2.25  (2.11, 2.39) | 0.92  (0.87, 0.97) |

5-year fold changes and ratios of 5-year fold changes were estimated using linear mixed models with random intercepts, with natural log (CACS+1) as the outcome. The model was adjusted for age, sex, traditional risk factors (hypertension, dyslipidemia, diabetes, smoking, alcohol consumption), BMI, and hsCRP. The L–H group was compared with the L–L group, and the H–L group was compared with the H–H group; L–L and H–H served as the respective reference groups.
Abbreviations: CAC: Coronary artery calcification, CI: Confidence interval, BMI: Body mass index, hsCRP: high-sensitivity C-reactive protein, TyG: Triglyceride-glucose.
